# Retraction notice to "Effect of derivatization method (KOH and BF3) on fatty acid profile data of boiled Tetracarpidium conophorum, and egusi pudding oils" [Data Brief 54 (2024) 110362]

**DOI:** 10.1016/j.dib.2024.111162

**Published:** 2025-03-25

**Authors:** Stephano Tambo Tene, Ronice Zokou, Paula Albendea, Leslie Gaddielle Demgne Bemmo, Giorgia Purcaro, Hilaire Macaire Womeni

**Affiliations:** a Research Unit of Biochemistry of Medicinal plants, Food Sciences and Nutrition, Department of Biochemistry, Faculty of Science, University of Dschang, P.O. Box 67 Dschang, Cameroon; b Gembloux Agro-Bio Tech, University of Liège, Passage des Déportés 2, Gembloux, 5030, Belgium; c Research Unit of Applied Botanic, Faculty of Science, University of Dschang, P.O. Box 67 Dschang, Cameroon

This article has been retracted: please see Elsevier Policy on Article Withdrawal (https://www.elsevier.com/about/policies/article-withdrawal).

The article has been retracted at the request of Dr. Albendea and Dr. Purcaro who were included in the author list of the paper. They have informed the journal that the corresponding author submitted this manuscript without their knowledge or approval. Moreover, these two persons do not support the content of the manuscript. One of the duties of the corresponding author is to ensure that all appropriate co-authors and no inappropriate co-authors are included on the paper, and that all co-authors have seen and approved the final version of the paper and have agreed to its submission for publication. For this submission, the corresponding author failed this duty, amongst others by registering incorrect email addresses for coauthors leading to them not receiving submission communications. *Data in Brief*, and Elsevier in general, follow the COPE guidelines for publishing ethics. The publication of this manuscript occurred with a clear breach of publishing ethics, leading to the contents of the manuscript being disputed by persons who are mentioned in the author list. The editorial board of *Data in Brief* offer their apologies to the readership of the journal for not detecting this issue during submission.

